# Supplementary material for: Novel Transcript Discovery Expands the Repertoire of Pathologically-Associated, Long Non-Coding RNAs in Vascular Smooth Muscle Cells
Source: Int J Mol Sci. 2021 Feb 2;22(3):1484. doi: 10.3390/ijms22031484 (PMC7867340; doi:10.3390/ijms22031484)
Supplement: Supplementary file 1 [file ijms-22-01484-s001.zip › ijms-1092837-supplementary/Supplementary Materials.pptx]

## Slide 1
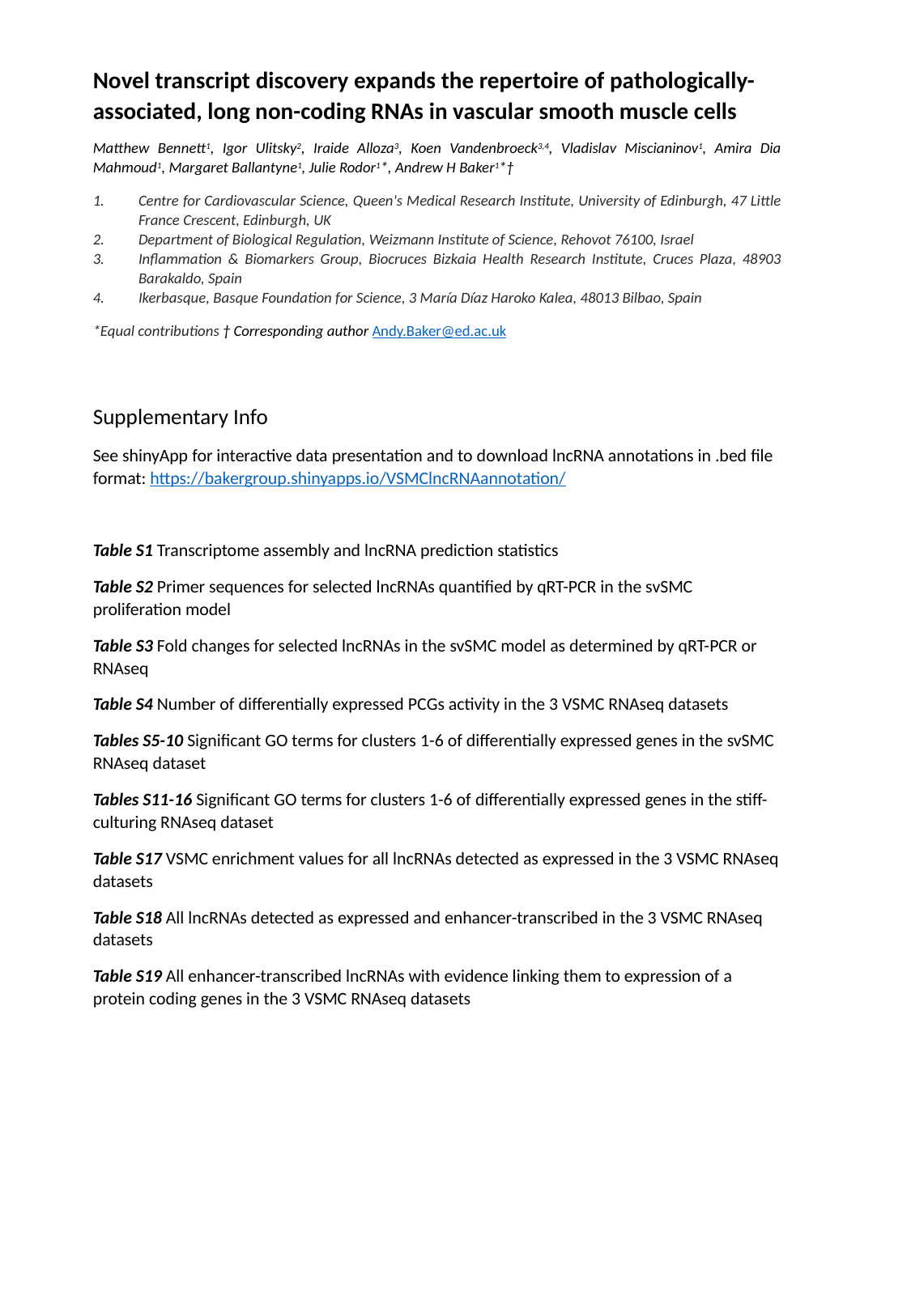

Novel transcript discovery expands the repertoire of pathologically-associated, long non-coding RNAs in vascular smooth muscle cells
Matthew Bennett1, Igor Ulitsky2, Iraide Alloza3, Koen Vandenbroeck3,4, Vladislav Miscianinov1, Amira Dia Mahmoud1, Margaret Ballantyne1, Julie Rodor1*, Andrew H Baker1*†
Centre for Cardiovascular Science, Queen's Medical Research Institute, University of Edinburgh, 47 Little France Crescent, Edinburgh, UK
Department of Biological Regulation, Weizmann Institute of Science, Rehovot 76100, Israel
Inflammation & Biomarkers Group, Biocruces Bizkaia Health Research Institute, Cruces Plaza, 48903 Barakaldo, Spain
Ikerbasque, Basque Foundation for Science, 3 María Díaz Haroko Kalea, 48013 Bilbao, Spain
*Equal contributions † Corresponding author Andy.Baker@ed.ac.uk
Supplementary Info
See shinyApp for interactive data presentation and to download lncRNA annotations in .bed file format: https://bakergroup.shinyapps.io/VSMClncRNAannotation/
Table S1 Transcriptome assembly and lncRNA prediction statistics
Table S2 Primer sequences for selected lncRNAs quantified by qRT-PCR in the svSMC proliferation model
Table S3 Fold changes for selected lncRNAs in the svSMC model as determined by qRT-PCR or RNAseq
Table S4 Number of differentially expressed PCGs activity in the 3 VSMC RNAseq datasets
Tables S5-10 Significant GO terms for clusters 1-6 of differentially expressed genes in the svSMC RNAseq dataset
Tables S11-16 Significant GO terms for clusters 1-6 of differentially expressed genes in the stiff-culturing RNAseq dataset
Table S17 VSMC enrichment values for all lncRNAs detected as expressed in the 3 VSMC RNAseq datasets
Table S18 All lncRNAs detected as expressed and enhancer-transcribed in the 3 VSMC RNAseq datasets
Table S19 All enhancer-transcribed lncRNAs with evidence linking them to expression of a protein coding genes in the 3 VSMC RNAseq datasets

## Slide 2
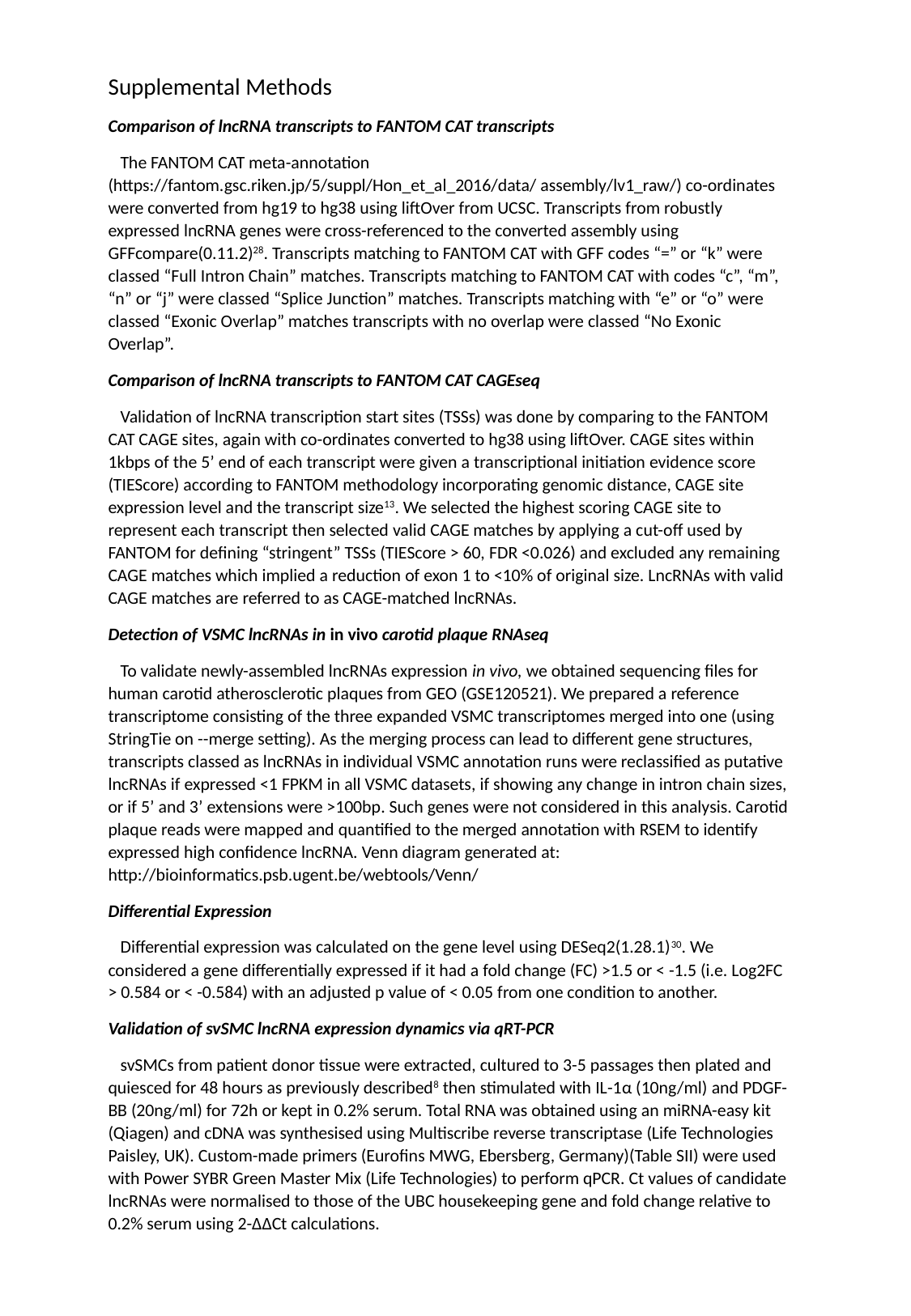

Supplemental Methods
Comparison of lncRNA transcripts to FANTOM CAT transcripts
 The FANTOM CAT meta-annotation (https://fantom.gsc.riken.jp/5/suppl/Hon_et_al_2016/data/ assembly/lv1_raw/) co-ordinates were converted from hg19 to hg38 using liftOver from UCSC. Transcripts from robustly expressed lncRNA genes were cross-referenced to the converted assembly using GFFcompare(0.11.2)28. Transcripts matching to FANTOM CAT with GFF codes “=” or “k” were classed “Full Intron Chain” matches. Transcripts matching to FANTOM CAT with codes “c”, “m”, “n” or “j” were classed “Splice Junction” matches. Transcripts matching with “e” or “o” were classed “Exonic Overlap” matches transcripts with no overlap were classed “No Exonic Overlap”.
Comparison of lncRNA transcripts to FANTOM CAT CAGEseq
 Validation of lncRNA transcription start sites (TSSs) was done by comparing to the FANTOM CAT CAGE sites, again with co-ordinates converted to hg38 using liftOver. CAGE sites within 1kbps of the 5’ end of each transcript were given a transcriptional initiation evidence score (TIEScore) according to FANTOM methodology incorporating genomic distance, CAGE site expression level and the transcript size13. We selected the highest scoring CAGE site to represent each transcript then selected valid CAGE matches by applying a cut-off used by FANTOM for defining “stringent” TSSs (TIEScore > 60, FDR <0.026) and excluded any remaining CAGE matches which implied a reduction of exon 1 to <10% of original size. LncRNAs with valid CAGE matches are referred to as CAGE-matched lncRNAs.
Detection of VSMC lncRNAs in in vivo carotid plaque RNAseq
 To validate newly-assembled lncRNAs expression in vivo, we obtained sequencing files for human carotid atherosclerotic plaques from GEO (GSE120521). We prepared a reference transcriptome consisting of the three expanded VSMC transcriptomes merged into one (using StringTie on --merge setting). As the merging process can lead to different gene structures, transcripts classed as lncRNAs in individual VSMC annotation runs were reclassified as putative lncRNAs if expressed <1 FPKM in all VSMC datasets, if showing any change in intron chain sizes, or if 5’ and 3’ extensions were >100bp. Such genes were not considered in this analysis. Carotid plaque reads were mapped and quantified to the merged annotation with RSEM to identify expressed high confidence lncRNA. Venn diagram generated at: http://bioinformatics.psb.ugent.be/webtools/Venn/
Differential Expression
 Differential expression was calculated on the gene level using DESeq2(1.28.1)30. We considered a gene differentially expressed if it had a fold change (FC) >1.5 or < -1.5 (i.e. Log2FC > 0.584 or < -0.584) with an adjusted p value of < 0.05 from one condition to another.
Validation of svSMC lncRNA expression dynamics via qRT-PCR
 svSMCs from patient donor tissue were extracted, cultured to 3-5 passages then plated and quiesced for 48 hours as previously described8 then stimulated with IL-1α (10ng/ml) and PDGF-BB (20ng/ml) for 72h or kept in 0.2% serum. Total RNA was obtained using an miRNA-easy kit (Qiagen) and cDNA was synthesised using Multiscribe reverse transcriptase (Life Technologies Paisley, UK). Custom-made primers (Eurofins MWG, Ebersberg, Germany)(Table SII) were used with Power SYBR Green Master Mix (Life Technologies) to perform qPCR. Ct values of candidate lncRNAs were normalised to those of the UBC housekeeping gene and fold change relative to 0.2% serum using 2-ΔΔCt calculations.

## Slide 3
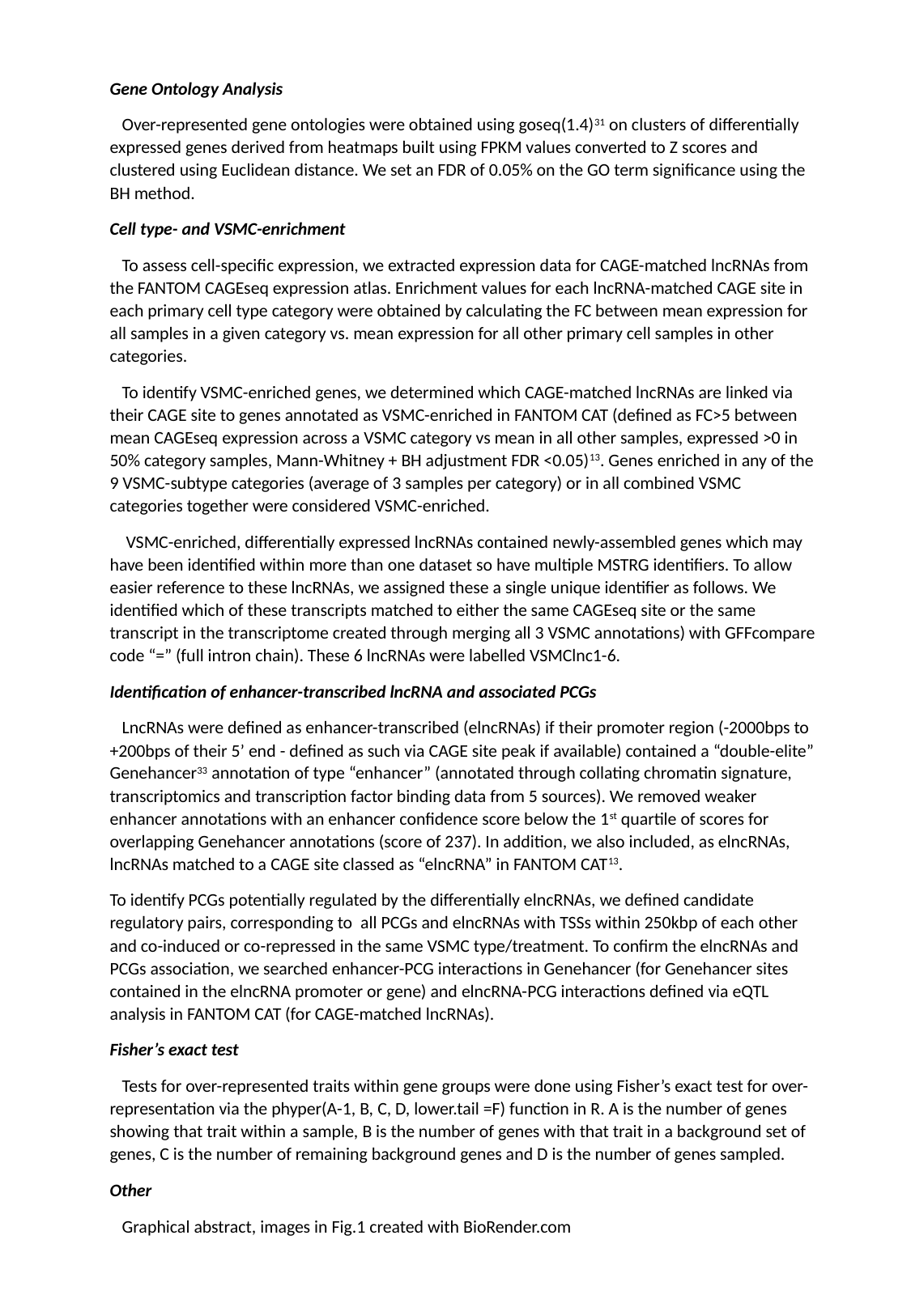

Gene Ontology Analysis
 Over-represented gene ontologies were obtained using goseq(1.4)31 on clusters of differentially expressed genes derived from heatmaps built using FPKM values converted to Z scores and clustered using Euclidean distance. We set an FDR of 0.05% on the GO term significance using the BH method.
Cell type- and VSMC-enrichment
 To assess cell-specific expression, we extracted expression data for CAGE-matched lncRNAs from the FANTOM CAGEseq expression atlas. Enrichment values for each lncRNA-matched CAGE site in each primary cell type category were obtained by calculating the FC between mean expression for all samples in a given category vs. mean expression for all other primary cell samples in other categories.
 To identify VSMC-enriched genes, we determined which CAGE-matched lncRNAs are linked via their CAGE site to genes annotated as VSMC-enriched in FANTOM CAT (defined as FC>5 between mean CAGEseq expression across a VSMC category vs mean in all other samples, expressed >0 in 50% category samples, Mann-Whitney + BH adjustment FDR <0.05)13. Genes enriched in any of the 9 VSMC-subtype categories (average of 3 samples per category) or in all combined VSMC categories together were considered VSMC-enriched.
 VSMC-enriched, differentially expressed lncRNAs contained newly-assembled genes which may have been identified within more than one dataset so have multiple MSTRG identifiers. To allow easier reference to these lncRNAs, we assigned these a single unique identifier as follows. We identified which of these transcripts matched to either the same CAGEseq site or the same transcript in the transcriptome created through merging all 3 VSMC annotations) with GFFcompare code “=” (full intron chain). These 6 lncRNAs were labelled VSMClnc1-6.
Identification of enhancer-transcribed lncRNA and associated PCGs
 LncRNAs were defined as enhancer-transcribed (elncRNAs) if their promoter region (-2000bps to +200bps of their 5’ end - defined as such via CAGE site peak if available) contained a “double-elite” Genehancer33 annotation of type “enhancer” (annotated through collating chromatin signature, transcriptomics and transcription factor binding data from 5 sources). We removed weaker enhancer annotations with an enhancer confidence score below the 1st quartile of scores for overlapping Genehancer annotations (score of 237). In addition, we also included, as elncRNAs, lncRNAs matched to a CAGE site classed as “elncRNA” in FANTOM CAT13.
To identify PCGs potentially regulated by the differentially elncRNAs, we defined candidate regulatory pairs, corresponding to all PCGs and elncRNAs with TSSs within 250kbp of each other and co-induced or co-repressed in the same VSMC type/treatment. To confirm the elncRNAs and PCGs association, we searched enhancer-PCG interactions in Genehancer (for Genehancer sites contained in the elncRNA promoter or gene) and elncRNA-PCG interactions defined via eQTL analysis in FANTOM CAT (for CAGE-matched lncRNAs).
Fisher’s exact test
 Tests for over-represented traits within gene groups were done using Fisher’s exact test for over-representation via the phyper(A-1, B, C, D, lower.tail =F) function in R. A is the number of genes showing that trait within a sample, B is the number of genes with that trait in a background set of genes, C is the number of remaining background genes and D is the number of genes sampled.
Other
 Graphical abstract, images in Fig.1 created with BioRender.com

## Slide 4
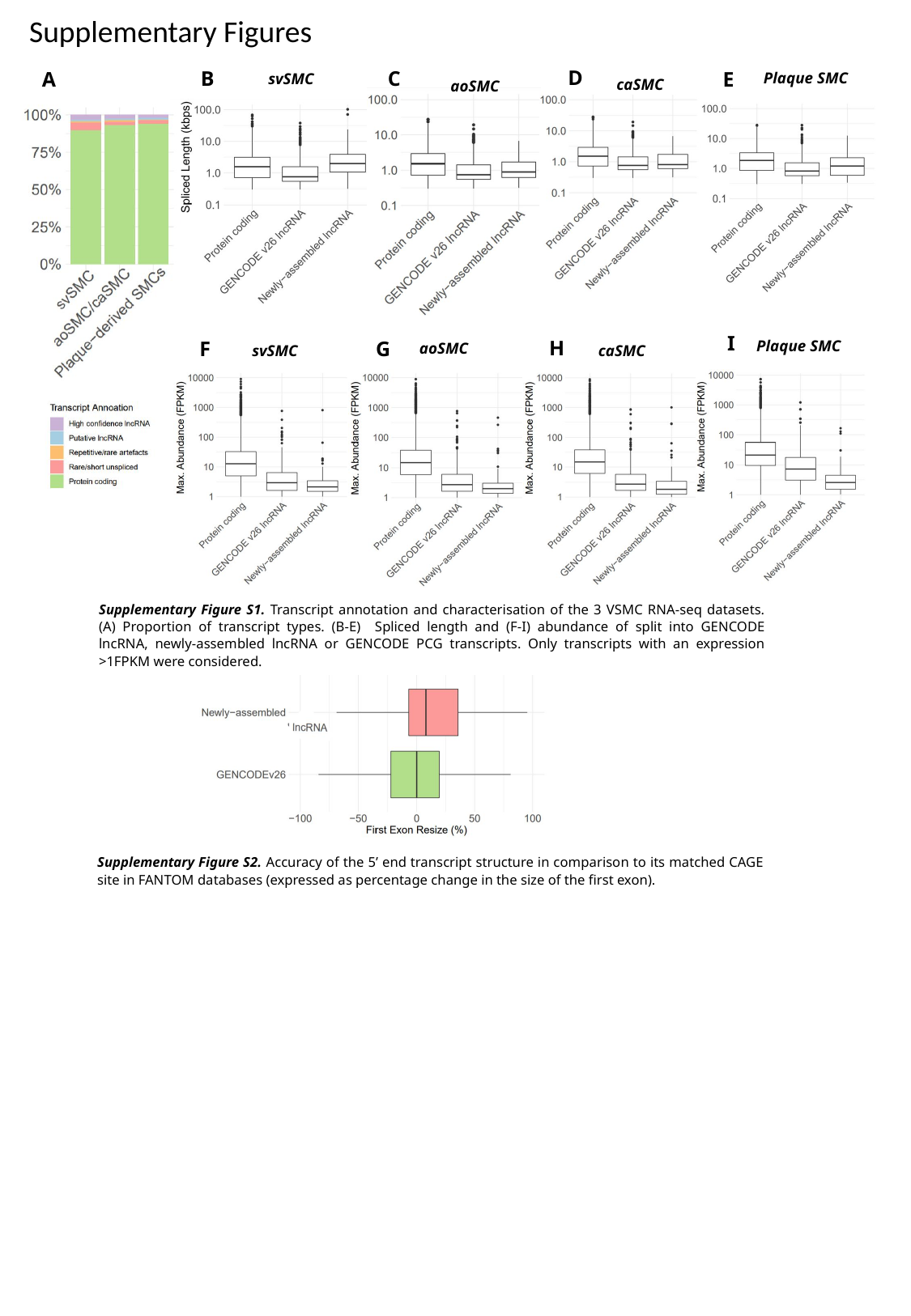

Supplementary Figures
D
C
B
A
E
Plaque SMC
svSMC
caSMC
aoSMC
I
H
F
G
Plaque SMC
aoSMC
svSMC
caSMC
Supplementary Figure S1. Transcript annotation and characterisation of the 3 VSMC RNA-seq datasets. (A) Proportion of transcript types. (B-E)   Spliced length and (F-I) abundance of split into GENCODE lncRNA, newly-assembled lncRNA or GENCODE PCG transcripts. Only transcripts with an expression >1FPKM were considered.
Supplementary Figure S2. Accuracy of the 5’ end transcript structure in comparison to its matched CAGE site in FANTOM databases (expressed as percentage change in the size of the first exon).

## Slide 5
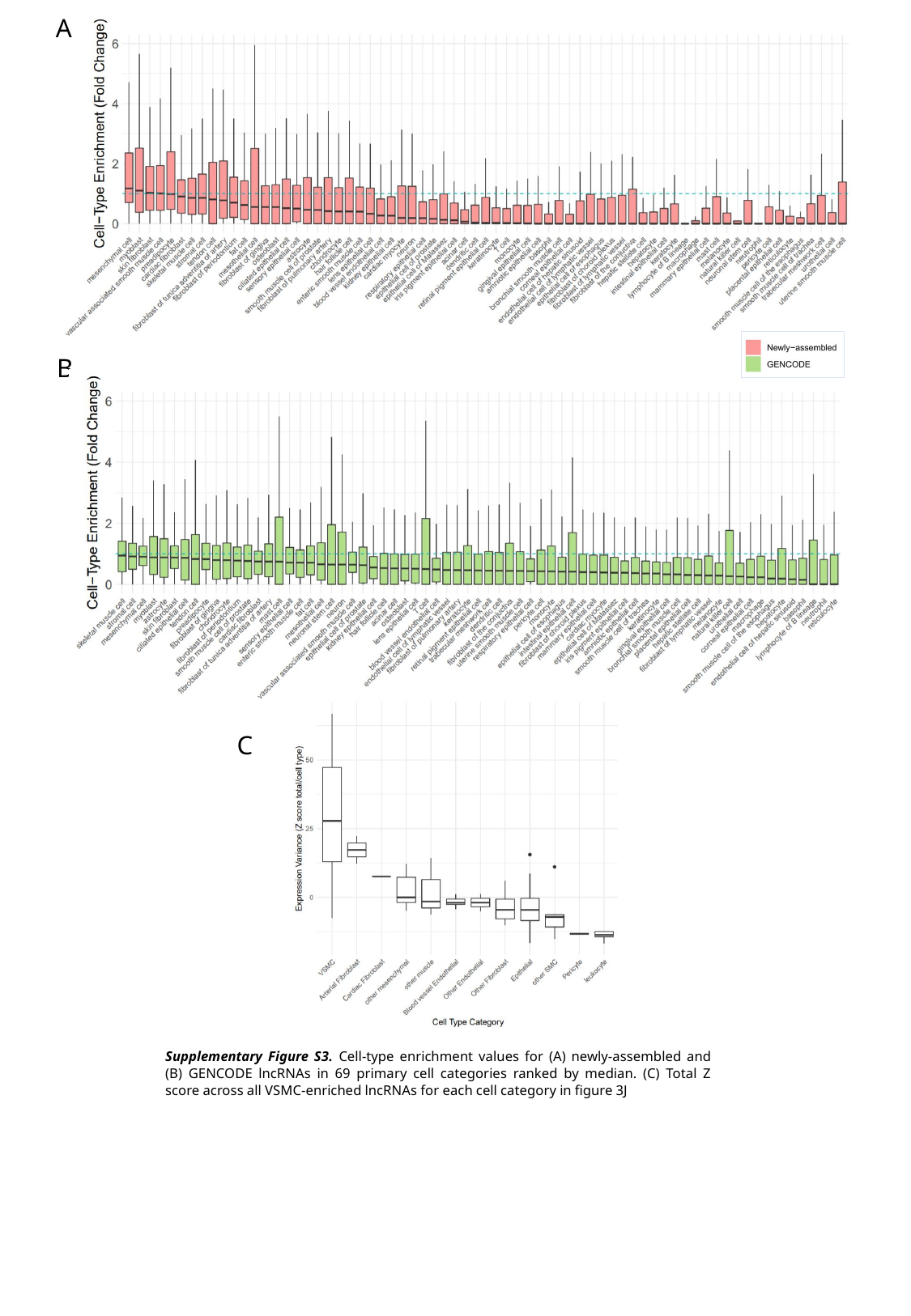

A
B
C
Supplementary Figure S3. Cell-type enrichment values for (A) newly-assembled and (B) GENCODE lncRNAs in 69 primary cell categories ranked by median. (C) Total Z score across all VSMC-enriched lncRNAs for each cell category in figure 3J

## Slide 6
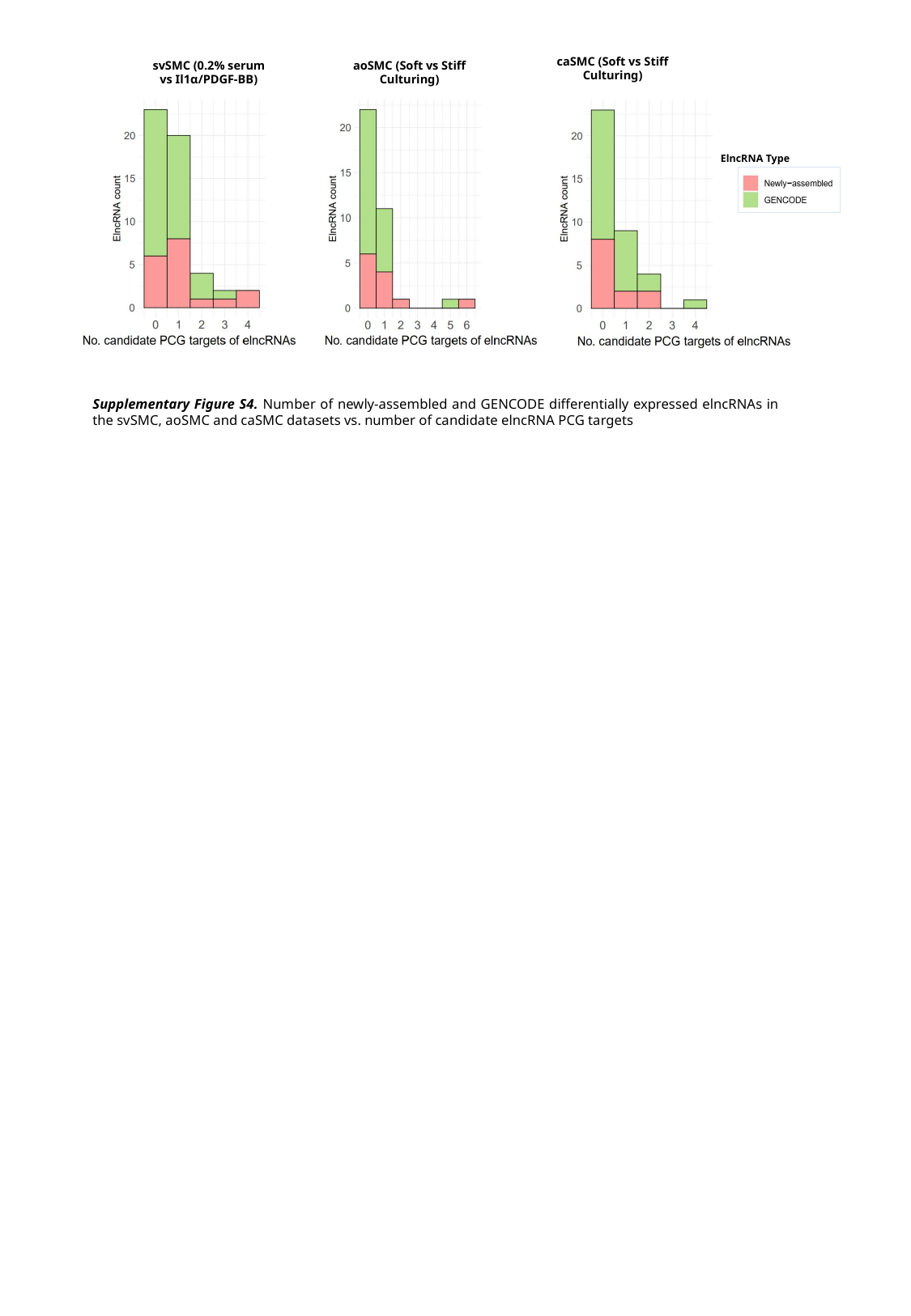

caSMC (Soft vs Stiff Culturing)
svSMC (0.2% serum vs Il1α/PDGF-BB)
aoSMC (Soft vs Stiff Culturing)
ElncRNA Type
Supplementary Figure S4. Number of newly-assembled and GENCODE differentially expressed elncRNAs in the svSMC, aoSMC and caSMC datasets vs. number of candidate elncRNA PCG targets
